# Postprandial effects of polydextrose on satiety hormone responses and subjective feelings of appetite in obese participants

**DOI:** 10.1186/1475-2891-14-2

**Published:** 2015-01-03

**Authors:** Kaisa Olli, Krista Salli, Esa Alhoniemi, Markku Saarinen, Alvin Ibarra, Tommi Vasankari, Nina Rautonen, Kirsti Tiihonen

**Affiliations:** DuPont Nutrition and Health, Active Nutrition, Sokeritehtaantie 20, FI-02460 Kantvik, Finland; Avoltus Oy, Turku, Finland; UKK Institute for Health Promotion Research, Tampere, Finland; Danisco Health and Nutrition, Kantvik, Finland

**Keywords:** Dietary fiber, GLP-1, Hunger, Lactate, Lactic acid, Obesity, Polydextrose, Satiety, VAS

## Abstract

**Background:**

Dietary fibers are associated with enhanced satiety. However, the mechanism of different dietary fibers contributing to satiety-related gastrointestinal (GI) peptide release, especially in an obese population, is still poorly understood. Polydextrose (PDX), a water-soluble glucose polymer, has demonstrated its ability to reduce energy intake at a subsequent meal, but its mechanism of action requires further research. Also, there is limited evidence on its capacity to regulate subjective feelings of appetite. This study examines the effects of PDX on postprandial secretion of satiety-related GI peptides, short chain fatty acids (SCFAs), lactic acid, and subjective appetite ratings in obese participants.

**Methods:**

18 non-diabetic, obese participants (42.0 y, 33.6 kg/m^2^) consumed a high-fat meal (4293 kJ, 36% from fat) with or without PDX (15 g) in an acute, multicenter, randomized, double-blind, placebo-controlled and crossover trial. Postprandial plasma concentrations of satiety-related peptides, namely ghrelin, cholecystokinin (CCK), glucagon-like peptide 1 (GLP-1), and peptide YY (PYY), as well as SCFAs and lactic acid were assessed. GI peptide, SCFA and lactate concentrations were then modeled using a linear mixed-effects model.

The subjective feelings of hunger, satisfaction, and desire to eat were evaluated using visual analogue scales (VAS), which were analyzed as incremental areas under the curve (iAUC) during the satiation and satiety periods.

**Results:**

We found that PDX supplementation increased plasma GLP-1 levels more than the placebo treatment (P = 0.02). In the whole group, GLP-1 concentrations found in participants older than 40 years old were significantly lower (P = 0.01) as compared to those aged 40 years or less. There were no statistically significant differences in postprandial ghrelin, CCK, or PYY responses. The lactic acid concentrations were significantly (P = 0.01) decreased in the PDX group, while no significant changes in SCFAs were found. PDX reduced iAUC for hunger by 40% (P = 0.03) and marginally increased satisfaction by 22.5% (P = 0.08) during the post-meal satiety period.

**Conclusion:**

Polydextrose increased the postprandial secretion of the satiety hormone GLP-1 and reduced hunger after a high-fat meal. PDX also reduced the elevated postprandial lactic acid levels in plasma. Therefore, PDX may offer an additional means to regulate inter-meal satiety and improve postprandial metabolism in obese participants.

## Background

Obesity is characterized by a chronic, low-grade inflammation that increases the prevalence of type 2 diabetes (T2D), metabolic syndrome, hypertension and cardiovascular disease [1, 2]. Adipokines released from the inflamed adipose tissue can cause insulin resistance and endothelial dysfunction [3]. An important link between obesity, metabolic syndrome and dyslipidemia appears to be the impaired ability of insulin to regulate glucose utilization in peripheral tissues [4].

Obesity is also known to affect postprandial metabolic processes and several implications of this effect have been reviewed recently [4–6]. Many obesity-associated metabolic risk factors, such as lipid abnormalities, may in fact be associated with the pro-inflammatory state initiated within the adipose tissue. Interestingly, changes in the concentrations of volatile short chain fatty acids (SCFAs) have been noted in obese subjects. In addition, the concentration of lactate seems to positively correlate with body weight [7] and the production of lactate in adipose tissue increases with the occurrence of obesity [7, 8].

Satiation and satiety are processes that are involved with the body’s appetite control system. Satiation leads to the termination of eating and it is accompanied by satisfaction of appetite. Satiety is explained as the feeling of fullness, which hinders hunger and further consumption of food. Satiation and satiety are both involved with limiting energy intake and thus it is important for determining the total energy intake. [9] Satiation and satiety are both influenced by energy density, macronutrient composition, physical structure and the sensory quality of ingested food [10]. If these two indicators of the subjective feelings of appetite could be regulated to the point where the intake of energy is significantly reduced, this would eventually lead to the overall control of body weight. In obese individuals, the ability of the gut to precisely monitor the luminal contents or relay this information back to the central nervous system is hindered [11]. An example is where the response of the vagal afferent neurons to the presence of gut peptides deteriorates, therefore diminishing its normal ability to inhibit food intake [11, 12].

Gastrointestinal peptides and metabolites can help us to understand the mechanism by which ingredients may affect food consumption and energy balance. Ghrelin is the first hormone to act in the appetite processes. It is involved in the meal initiation and peak levels of ghrelin are recorded just prior to food consumption [13]. Short-term signals that are released during, or between, meals include gastrointestinal (GI) peptides such as cholecystokinin (CCK), glucagon-like peptide 1 (GLP-1), and peptide tyrosine tyrosine (PYY) [14]. The gut hormone CCK appears to be involved in satiation [14] and its experimental administration reduces subsequent meal size [15]. However, CCK’s effect on suppressing food intake is enhanced by stomach distension [16]. GLP-1 is a potential biomarker for satiety [14]. When administered experimentally to humans it can suppress energy intake, promote satiety and decrease hunger [17, 18]. There is also evidence showing reduced GLP-1 levels in obese subjects [19]. In addition, the administration of PYY, another GI peptide affecting satiety, has been reported to reduce food intake in humans [20]. CCK, GLP-1, and PYY are released by the enteroendocrine I and L cells of the small and large intestine in response to food intake, i.e. during a meal [14, 21]. However, the release of PYY begins even before the nutrients reach the part of the gut where it is produced [22]. These peptides are involved with the process which helps to regulate the intestinal secretion and gut motility by transporting signals to the brain which then reduce appetite [14, 23].

Different foods have varying effects on postprandial metabolism and more specifically on the secretion of gut peptides [24]. Generally, fibers in food are able to enhance satiety by adding bulk and viscosity [25]. They can also alter the secretion of gut hormones and, thus, influence metabolism and energy expenditure [26]. The impact of different non-starch polysaccharides on satiety and satiation have recently been reported [27]. In addition, previous studies on prebiotic fibers carried out on humans and animals have shown that the consumption of lactitol [28] and other highly fermentable fibers [29, 30] is able to stimulate the secretion of GLP-1. On the other hand, increased concentrations of plasma PYY have also been reported following the ingestion of lactitol [28] and inulin [31].

Polydextrose (PDX) is a randomly bonded, soluble and branched glucose polymer with a high molecular weight and low colonic fermentation rate [32, 33]. PDX has been recognized as soluble fiber and several studies have demonstrated beneficial physiological effects associated with this feature [34]. PDX is approved for use in foods in over 60 nations and recognized as a dietary fiber in more than 20 countries [35]. In addition, PDX is widely used as a sugar and fat replacement in various food products [36, 37]. PDX has a low energy-density (4 kJ/g) and has been shown to increase satiety and reduce energy intake during a subsequent meal when administered as a supplement 1.5 to 1 h before *ad libitum* lunch [38–41]. However, this effect was not significant when PDX was given as a part of a breakfast meal [42, 43]. PDX was recently demonstrated to reduce the desire to eat and the feeling of hunger when it replaced 30% of the other available carbohydrates in the diet [44] equivalent to a supplement of almost 50 g of PDX per day. But when lower concentrations of PDX were used the results on appetite ratings were not consistent [41, 42, 45]. However, PDX can help maintain low postprandial blood glucose levels [46].

Even though PDX has demonstrated to reduce energy intake during a subsequent meal, its mechanism of action is not yet fully understood. This study examines the effects of a PDX-supplemented meal on appetite regulation in obese participants. There are several studies showing a reduction in appetite following the ingestion of PDX as reported in both normal weight and over-weight volunteers [39–41], however the data on obese participants is lacking. The appetite suppressing mechanism of PDX is hypothesized to function through satiety hormones. Since satiety can also be linked to intestinal fermentation [47, 48], the postprandial plasma concentrations of lactate and SCFAs were also evaluated.

## Methods

### Participants

The protocol was approved by the Research Ethics Committee from the Hospital District of Northern Savo in Finland (123/2007). The study was conducted in 2008 in two Finnish research centers located in Kuopio and Vierumäki following the guidelines laid down by the Declaration of Helsinki. The purpose of the study was explained to all participants who gave their written informed consent to be included in the study.

The inclusion criteria for participants were as follows: age between 20 to 55 years old, body mass index (BMI) between 30 to 37 kg/m^2^, and non-diabetic. In addition, PDX is considered to be a dietary fiber [34]; hence the participants had to have adapted a diet with moderately low fiber content (typical fiber intake of less than 19 g/day for men and 17 g/day for women). Participants were excluded if they presented any critical illness, inflammatory bowel disease, celiac disease or malignancy in the GI track, pregnancy, cardiovascular or metabolic diseases, or if they were using any lipid lowering medication affecting serum triglyceride concentrations, anti-obesity drugs or dietary supplements with a high fiber content. Furthermore, the regular (daily) use of fiber supplements, bran or seeds as well as the regular and abundant use of non-steroidal anti-inflammatory drugs was not allowed. In addition, persons with familial hyperlipidemia were excluded from the study. A structured interview focusing on previous and current diseases, current medication, and alcohol and tobacco consumption was carried out during a screening process to clarify the health status and suitability of the participants. Body weight and height were also measured and fasting blood samples were taken during this screening process.

### Study design

An acute, standardized, postprandial, randomized, double-blind, placebo-controlled, cross-over (10 days wash-out) and multicenter study using a high-fat meal (4293 kJ, 36% from fat) was conducted on 18 volunteers according to a similar protocol as described earlier by Ahotupa et al. [49]. The primary outcome of this study was to determine any postprandial changes in serum triglycerides (Tiihonen et al., unpublished observations). The secondary outcomes assessed and reported in this article were the effects of polydextrose on the satiety hormone levels and subjective feelings of appetite using VAS scores.

The participants included in this acute study were selected from a related 4-week intervention study (not reported here). Each participant was examined on two separate occasions. Their body weight and height were determined during the initial screening. The participants were asked not to alter their medication, lifestyle or body weight during the study and were advised to avoid strenuous exercise and not to consume alcohol for 24 h before the test days, nor to eat any items rich in fat on the day before the test days. Participants’ use of nicotine-containing products was noted from 24 h prior to the start of the study. They were allowed to consume up to 10 cigarettes (or equivalent) per day. Participants also recorded their dietary intake from 3 p.m. the day before the first postprandial test day and were asked to adhere to this diet prior to the second postprandial test to ensure a standardized meal load for both sessions.

After a 10 to 12 h fasting period on the study day, a high-fat meal was provided to the participants in a randomized order. The intervention meals were served at 10 a.m. and the last blood samples were taken at 4.30 p.m. Participants were asked to eat the meal in 20 minutes and they were not allowed to consume any additional food during the 6 h testing period, except for water. Participants spent the study days in the laboratory and all physical exercise was forbidden during that time.

### Composition of the study meal

The study meal consisted of a standard commercial hamburger, french-fries and a carbonated drink. The energy and nutrient content of the study meal is presented in Table 1. The experimental meals contained either 15 g of PDX (Litesse® Ultra™, DuPont) or not (placebo) incorporated into the drink. The amount of PDX used in this study was aligned with the effective doses reported in previous clinical trials [39, 41]. The placebo drink contained no additional ingredients. The addition of PDX to the test drink increased the energy content of the meal only by 60 kJ. A previous blind sensory test ensured that PDX did not change the appearance or taste of the drink.

**Table 1:** **The energy and nutrient content of the study meals in placebo and PDX groups** [50]

	Weight (g)/volume (mL)	Energy (kJ)	Protein (g)	Carbohydrates (g)	Fat (g)	Fiber (g)	Salt (g)
**Hamburger**							
Both groups	219	2071	27	40 (incl. 8 g sugar)	25 (SFAs 10 g)	3	2.3
**French fries**							
Both groups	114	1423	5	42 (incl. 1 g sugar)	17 (SFAs 3 g)	4	0.4
**Carbonated drink**							
Placebo group	450	799	< 0.1	42	< 0.1	0	0
PDX group	450	799	< 0.1	42	< 0.1	15	0
**Total**							
Placebo group	783	4293	32	124	42	7	2.7
PDX group	783	4353	32	124	42	22	2.7

PDX, polydextrose; SFA, saturated fatty acid.

The meal termination time was the point at which the whole meal had been consumed. Two hundred mL of water was served to the participants two and four hours after the meal. Additional spices were not allowed during the meal.

### Blood sampling and analysis

Fasting blood samples used to analyze the lipid profile, plasma glucose and serum insulin concentrations were taken during the initial screening. To determine the concentration of ghrelin, CCK, GLP-1, PYY, SCFAs and lactate, venous blood samples (10 mL) were taken through a cannula inserted in a forearm vein twice before the meal (0-sample), and 60, 120, 240 and 360 minutes after the beginning of the meal during both study periods. A baseline was set up with two measurements (0-samples) taken within 10 minutes of each other. Serum was separated by centrifugation (3000 rpm, 10 to 15 min) after which it was stored at –70°C until analyzed. Plasma samples were frozen at –70°C until analyzed.

The total plasma cholesterol content was analyzed using an enzymatic, photometric method. High and low density lipoprotein (HDL and LDL, respectively) cholesterols were analyzed by direct measurement and the total triglyceride concentrations were analyzed by enzymatic colorimetric assay using an automatic analyzer (Roche/Hitachi MODULAR ANALYTICS, Roche Diagnostics GmbH, Mannheim, Germany) and commercial reagents (cholesterol CHOD-PAP, Cat. No. 11875540; HDL-cholesterol, Cat. No. 04713214; LDL-cholesterol, Cat. No. 03038777; triglycerides, GPO-PAP, Cat. No. 11730711, Roche Diagnostics GmbH, Mannheim, Germany). The plasma LDL-cholesterol concentration was calculated using the Friedewald formula [51]. Plasma glucose concentrations were analyzed using the hexokinase method with citrate-fluoride. The serum insulin concentrations were determined using an immunoluminometric assay measured with an Immulite 2000 Analyzer (Thermo Fisher Scientific Inc.).

The GI peptide (ghrelin, CCK, GLP-1, and PYY) concentrations in the plasma samples were analyzed by competitive enzyme immunoassays (Phoenix Pharmaceutical, Burlingame, CA, USA) according to the assays’ manufacturer’s instructions.

Plasma SCFAs (acetic acid, propionic acid, butyric acid, isobutyric acid, valeric acid, isovaleric acid, and 2-methylbutyric acid) and lactic acid were analyzed by gas chromatography as follows: internal standard (25 μL 20 mM pivalic acid) and 1.5 mL of acetonitrile were added to 0.5 mL of the plasma sample. The sample was then mixed thoroughly and centrifuged at 16000 × g for 3 min. The supernatant was then transferred to a 2 mL centrifuge tube and 25 μL of 0.2 M NaOH was added. After mixing, the sample was heated in a vacuum centrifuge at 60°C until dry. The residue was re-dissolved in 100 μL of saturated oxalic acid–water solution (37:63). Then, 1 μL of the sample solution was analyzed by gas chromatography using a glass column packed with 80/120 Carbopack B-DA/4% Carbowax 20 M stationary phase (2 m × 2 mm, Supelco, Bellefonte PA, USA) at 175°C and using helium as the carrier gas at flow rate of 24 mL/min. The temperature of the injector and the flame ionization detector were 200°C and 245°C, respectively.

### Appetite ratings

Participants reported their subjective feelings of appetite at time-point 0 (prior to the consumption of the meal) and 40, 70, 140 and 280 minutes after the initiation of the study meal. They rated their appetite feelings using 100 mm visual analogue scales (VAS) anchored at both extremes, which were presented in paper form. Three appetite ratings were evaluated in the study: hunger (*How hungry do you feel at the moment? I am not at all hungry – I am very hungry*), satisfaction (*How satisfied do you feel at the moment? I do not feel satisfied at all – I feel very satisfied*), and desire to eat (*How strong is your desire to eat at the moment? I do not have desire to eat at all – My desire to eat is very strong*). These questions were adapted from [52] and [53]. The participants made a vertical mark between the two extremities at the place corresponding to what they were feeling at the exact moment when the VAS was presented. The scores for appetite ratings were obtained by measuring the interval between the extreme left and the mark made by the participant.

### Statistical analyses

#### Baseline characteristics

The similarity of the groups for the continuous-valued parameters was assessed using the Student’s *t* test (normally distributed parameters) or Mann-Whitney U test (other parameters). For the discrete-valued gender parameter, Fisher’s exact test was used.

#### GI peptides, SCFAs and lactate

The results showing GI peptide, SCFA and lactate concentrations are presented here using an exploratory statistical model. The concentrations were modeled using a linear mixed-effects model which had fixed effect terms for treatment, time point and interaction between treatment and time point, as well as covariates for baseline value, gender, BMI and age - i.e. the models were adjusted for these factors. In addition, each model had a random effect, which is a subject-wise intercept term that takes into account the fact that a setting with repeated measures per subject was used. Post-hoc comparisons for GI peptides and lactic acid were carried out for statistically significant model terms of interest, e.g. age, BMI, or treatment. The comparisons were carried out using model contrasts and if multiple hypotheses were tested, the *p* values were adjusted to avoid false positives. If the model terms of interest of BMI and age were significant, further post-hoc analyses were conducted considering the following cut-off values: BMI ≤ 34 kg/m^2^ and BMI > 34 kg/m^2^; and age ≤ 40 y and age > 40 y.

Distribution of data values have been presented separately for each treatment and time point using box plots. The whiskers represent minimum and maximum values of the data (exceptionally large or small values outside this range are shown using black dots). The box borders indicate the 25% and 75% percentiles of the data and the line inside the box is median of the data. As presented in the figures, values in the y-axis are the original values that have been subtracted from the treatment-wise median of values at time point 0. Vertical shifting of the curves was done in order to ensure that their medians are 0 at time point 0.

The linear mixed-effects model analyses were carried out using R: A Language and Environment for Statistical Computing ver. 3.01 [54]. The models were computed using package nlme ver. 3.1 [55] and the contrasts were computed using package multcomp ver. 1.2 [56].

#### Appetite ratings

Appetite ratings of hunger, satisfaction, and desire to eat were divided into two periods, namely ‘satiation’ and ‘satiety’, and expressed as incremental areas under the curve (iAUC). To this end, appetite rating curves were adjusted to zero intensity at time point 40 min (post meal). Thus, iAUC for satiation corresponds to the time points starting immediately before the meal (0 min) and continuing 40 minutes after the initiation of the meal; and iAUC for satiety corresponds to the period between the finalization of the meal (40 min) and the end of the appetite ratings assessment (280 min). iAUC are reported as mean ± SEM in min.mm. iAUC results for satiation and satiety periods of each appetite rating for PDX or placebo were compared using Student’s *t* tests (paired, two-tailed).

#### Sample size

Since the primary outcome of this clinical study was to determine postprandial changes in serum triglycerides (not reported here), the sample size was estimated according to this parameter. Therefore, the sample size of 18 participants was calculated to be able to detect 86 mmol/L × min difference in iAUC for serum triglyceride concentration between the study periods with 80% power at α-level of 0.05. The assumed standard deviation for iAUC for serum triglyceride concentration was 130 mmol/L × min.

Equivalent sample sizes have been used in similar study designs investigating changes in gastric peptides [24, 28, 57, 58].

## Results

### Characteristics of the study participants

Thirteen females and five males participated in the study. The demographics and clinical characteristics of the participants are presented in Table 2. These parameters are referential values from the 4-week intervention that were taken at screening. The mean BMI of study participants was 33.6 (min 30 – max 37) and the mean age 42 (±8) years. There were nine participants with a BMI exactly or less than 34 kg/m^2^ and nine participants with a BMI higher than 34 kg/m^2^. With regards to age, seven participants were younger or 40 years old and eleven participants were older than 40 years. Although the participants were clinically obese, their glucose and lipid metabolism appeared not to be significantly disturbed. The average serum triglycerides were slightly above the normal level and the total cholesterol was at borderline of high risk according to the American Heart Association’s guidelines [59]. However, the serum concentrations of LDL and HDL-cholesterol were near optimal or medium levels, respectively [59].

**Table 2:** **The demographic and clinical characteristics of the study participants**

Characteristic	
Men/women*	5/13
Age (years)	42.0 (26–53)
Weight (kg)	94.8 (73–114)
BMI (kg/m^2^)	33.6 (30–37)
Glucose (mmol/L)	5.65 ± 0.58
Insulin (mU/L)	9.75 ± 6.81
Triglycerides (mmol/L)	1.71 ± 0.96
Total cholesterol (mmol/L)	5.28 ± 1.19
LDL-cholesterol (mmol/L)	3.12 ± 0.96
HDL-cholesterol (mmol/L)	1.43 ± 0.51

Values are expressed as ratios*, mean values with their ranges (min–max) or standard deviations (±SD).

BMI, body mass index; HDL, high density lipoprotein; LDL, low density lipoprotein.

The population randomly selected into two arms was homogenous, except for weight, which differed slightly (P = 0.04) between the two populations. However, this did not affect the results of this study, since weight gain was not followed here. Additionally, there were no statistical differences between the baseline values for satiety peptides in the placebo and PDX groups.

### Gastrointestinal peptides

**Figure 1:**
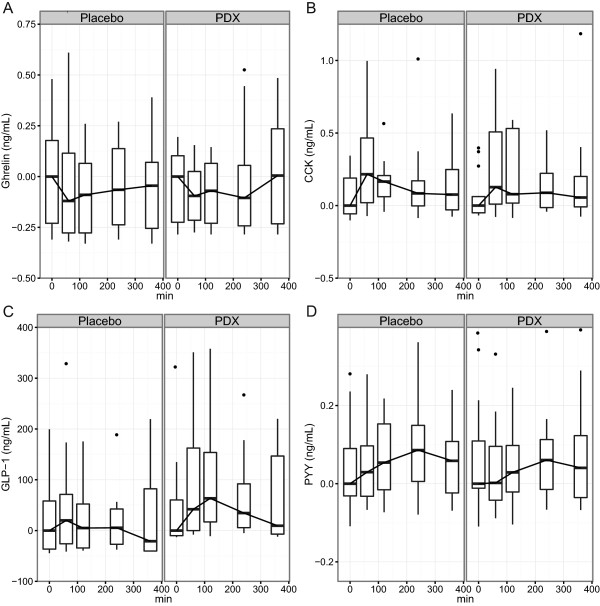
**Changes in the concentrations of gastrointestinal peptides in plasma.** Changes in ghrelin **(A)**, CCK **(B)**, GLP-1 **(C)** and PYY **(D)** after the ingestion of the study meal supplemented with the placebo or polydextrose (PDX) presented as box plots and analyzed by a linear mixed-effects model. The GLP-1 response was increased significantly (P = 0.02) with PDX when compared to the placebo. The concentrations of ghrelin, CCK, and PYY were not significantly altered by the treatments. The median of all values at the first time point (0 min) has been subtracted from the parameter values presented in the box plots. CCK, cholecystokinin; GLP-1, glucagon-like peptide 1; PYY, peptide YY.

The kinetic parameters of GI peptides are presented in Table 3. Baseline values were statistically significant factors (P < 0.0001) explaining the differences in the responses of CCK, GLP-1, and PYY. Of all the GI peptides that were monitored, only the secretion of GLP-1 was significantly affected (P = 0.006) by the treatment effect; placebo vs. PDX. In addition, GLP-1 and CCK concentrations were affected by the time (P < 0.01). Furthermore, both age and BMI significantly affected the concentration of GLP-1 (P = 0.02 and P = 0.04, respectively) and therefore a post-hoc test was carried out. When these factors were taken into consideration within a post-hoc multiple-hypothesis test, it was observed that GLP-1 secretion into the plasma increased significantly (P = 0.02) after the consumption of PDX compared to the placebo (Figure 1C).

**Table 3:** **Kinetic parameters of gastrointestinal peptides**

	Ghrelin		CCK		GLP-1		PYY	
	Placebo	PDX	Placebo	PDX	Placebo	PDX	Placebo	PDX
Baseline (ng/mL)	0.41 ± 0.26	0.32 ± 0.17	0.33 ± 0.32	0.24 ± 0.15	130.8 ± 151.4	143.6 ± 218.4	0.43 ± 0.11	0.47 ± 0.14
C_max_ (ng/mL)	0.54 ± 0.31	0.52 ± 0.35	0.8 ± 1.21	1.14 ± 1.64	212.7 ± 269.5	226.1 ± 229.6	0.53 ± 0.14	0.56 ± 0.19
T_max_ (min)	100 ± 122	163 ± 160	120 ± 101	147 ± 113	157 ± 135	177 ± 119	173 ± 107	163 ± 139

Values are shown as means ± standard deviations.

CCK, cholecystokinin; GLP-1, glucagon-like peptide 1; PDX, polydextrose; PYY, peptide YY.

Because the model terms of interest of BMI and age were statistically significant in the GLP-1 model, a further post-hoc analysis was conducted with selected cut-off values in the whole group. Results show that concentrations of GLP-1 in participants older than 40 years old were significantly lower (P = 0.01) as compared to those aged 40 years or less. However, there were no significant differences between participants with a BMI that was higher or lower than 34 kg/m^2^ (P = 0.1).There were no statistically significant differences in the concentrations of ghrelin, CCK or PYY (Figure 1A, B and D) between the placebo and the PDX test meals when analyzed by a linear mixed-effects model. However, there was a tendency for the concentrations of ghrelin and PYY to decrease after the consumption of PDX, but the values did not reach statistically significant levels due to a large standard deviation (P = 0.06 and P = 0.08, respectively).

### Plasma SCFAs and lactic acid

The kinetic parameters of acetic acid and lactic acid are presented in Table 4. The baseline values were statistically significant factors (P < 0.0001) which explain the differences in the responses of acetic acid and lactic acid. All other SCFAs were below detection limits. Acetate and lactate exist in an ionized form within the usual plasma pH range (7.35–7.45), thus they were analyzed in the form of acetic acid and lactic acid.

**Figure 2:**
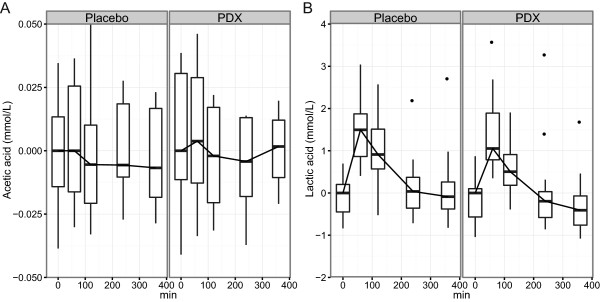
**Changes in the concentrations of acetic acid and lactic acid in plasma.** Changes in postprandial acetic acid **(A)** and lactic acid **(B)** in plasma after the ingestion of the study meal supplemented with the placebo or polydextrose (PDX) presented as box plots and analyzed by a linear mixed-effects model. The decrease (11.9%) in the concentration of lactic acid after the consumption of PDX was statistically significant when compared to the placebo (P = 0.01). The curves are adjusted to zero by subtracting the median of all values at the first time point (0 min) from the parameter values presented in the box plots.

A marginal 14.3% reduction in the concentration of acetic acid after the consumption of PDX was observed (P = 0.07) when analyzed by a linear mixed-effects model (Figure 2A). The concentration of lactic acid was significantly (P = 0.01) affected by the consumption of PDX when compared to the control treatment. A linear mixed-effects model with a post-hoc comparison for a treatment effect shows that the concentration of lactic acid decreased by 11.9% after the consumption of PDX (Figure 2B). In addition, the time-point had a statistically significant (P < 0.0001) effect on the differences in lactic acid concentrations.

**Table 4:** **Kinetic parameters of acetic acid and lactic acid**

	Acetic acid		Lactic acid	
	Placebo	PDX	Placebo	PDX
Baseline (mmol/L)	0.12 ± 0.1	0.16 ± 0.22	1.52 ± 0.44	1.79 ± 1.23
C_max_ (mmol/L)	0.17 ± 0.14	0.17 ± 0.22	3.39 ± 1.35	3.32 ± 1.09
T_max_ (min)	127 ± 142	80 ± 105	83 ± 72	67 ± 46

Values are shown as means ± standard deviations.

PDX, polydextrose.

### Appetite ratings

**Figure 3:**
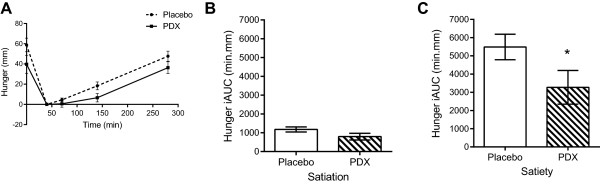
**VAS results for the subjective feelings of hunger. A)** The VAS curves of the placebo and polydextrose (PDX) adjusted to zero intensity at 40 min (after the meal). **B)** The incremental areas under the curve (iAUC) for the placebo and PDX during the satiation period, i.e. between 0 min and 40 min (1176 ± 135 min.mm and 794 ± 179 min.mm, respectively, P > 0.05). **C)** The iAUC for the placebo and PDX during the satiety period, i.e. between 40 min and 280 min, differed statistically (5487 ± 700 min.mm and 3272 ± 923 min.mm, respectively, P < 0.05*). VAS, visual analogue scales.

**Figure 4:**
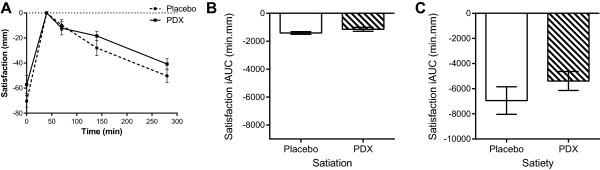
**VAS results for the subjective feelings of satisfaction. A)** The VAS curves of the placebo and polydextrose (PDX) adjusted to zero intensity at 40 min (after the meal). **B)** The incremental areas under the curve (iAUC) for the placebo and PDX during the satiation period, i.e. between 0 min and 40 min (-1409 ± 95 min.mm and -1144 ± 143 min.mm, respectively, P > 0.05). **C)** The iAUC for the placebo and PDX during the satiety period, i.e. between 40 min and 280 min (-6944 ± 1095 min.mm and -5385 ± 735 min.mm, respectively, P = 0.08). VAS, visual analogue scales.

**Figure 5:**
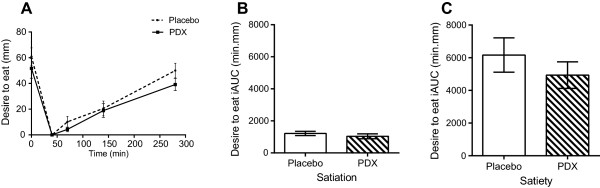
**VAS results for the subjective feelings of desire to eat. A)** The VAS curves of the placebo and polydextrose (PDX) adjusted to zero intensity at 40 min (after the meal). **B)** The incremental areas under the curve (iAUC) for the placebo and PDX during the satiation period, i.e. between 0 min and 40 min (1218 ± 134 min.mm and 1034 ± 153 min.mm, respectively, P > 0.05). **C)** The iAUC for the placebo and PDX during the satiety period, i.e. between 40 min and 280 min (6167 ± 1052 min.mm and 4939 ± 814 min.mm, respectively, P > 0.05). VAS, visual analogue scales.

Results on appetite ratings are presented for hunger (Figure 3), satisfaction (Figure 4), and the desire to eat (Figure 5). There were no significant differences on the satiation effects for the three appetite ratings tested (Figures 3B, 4B and 5B). PDX significantly (P = 0.03) reduced hunger by 40.4% as compared to the placebo during the satiety period (Figure 3C). In addition, PDX marginally (P = 0.08) increased satisfaction by 22.5% as compared to the placebo during the same period (Figure 4C). There were no statistically significant differences on the subjective feelings of desire to eat during the satiety period (Figure 5C).

## Discussion

Dietary fibers are known to be associated with an enhanced satiety. However, the mechanism by which the different dietary fibers contribute to the satiety-related GI peptide release, especially in an obese population, is still poorly understood. PDX shows similar beneficial gastrointestinal functionalities as conventional grains and thus fulfils the definition of dietary fiber in this respect [34]. However, there are not enough data to conclude the effects of PDX on postprandial blood glucose and fasting cholesterol levels [34]. As hypothesized in this study, the potential for dietary PDX to affect the postprandial metabolic parameters in obese subjects was evaluated here with significant results in GLP-1 secretion, plasma lactate concentration and the subjective feelings of hunger. The hypothesis was based on the observations found in the preliminary study conducted with obese participants [60].

In this study, the obese - but otherwise healthy - participants consumed a typical western, moderately low fiber diet. A high-fat hamburger meal was used as a model and it has been reported to function successfully as a postprandial lipaemia model [49]. Here, PDX was added to a carbonated beverage consumed simultaneously with the study meal. This presented a practical and efficient way to increase the fiber content of a meal as well as to study the postprandial effects of fiber-enrichment.

Different fermentable dietary fibers have previously been reported to increase GLP-1 concentrations in animal studies [29, 61, 62], however similar results from human studies conducted with PDX had been few until now, or reported in combination with other satiating enhanced products [63]. In the present study, supplementing a high-fat meal with dietary PDX significantly increased the concentration of postprandial plasma GLP-1, when compared to the placebo. The GLP-1 response 120 min after PDX supplementation correlates kinetically with the intestinal fermentation pattern of PDX as measured by breath hydrogen [64]. The most important metabolic actions of GLP-1 are the stimulation of insulin secretion and inhibition of glucagon secretion; both of which are considered glucose-dependent [65]. Native GLP-1 has a short half-life, but its longer-acting analogues have been used successfully in the treatment of obesity and T2D [65], and GLP-1 has been shown to decrease blood glucose in human subjects with T2D (reviewed in [66]). In the GI tract, GLP-1 can slow down the postprandial gastric emptying rate and promote satiety through interactions with the gut and brain [65]. Hence, the increased postprandial GLP-1 observed in this study can present one mechanistic explanation for the capability of PDX to increase satiety [39].

One of the most striking results of our study was to find that age might also be an important factor in the GLP-1 response. Here we report lower GLP-1 concentrations in participants older than 40 years old when compared to those aged 40 years or less in the whole group of obese people. It is known that aging impairs insulin sensitivity and reduces insulin and incretin production [67]. In healthy subjects GLP-1 plays a key role in the postprandial process which leads to the secretion of insulin. However, this incretin effect is diminished in subjects with T2D and those with impaired glucose tolerance. [68] Postprandial GLP-1 and PYY responses have also been shown to be impaired in obese subjects compared to those of normal weight [19, 20].

The effect of PDX on postprandial ghrelin, CCK or PYY release had not previously been reviewed in obese subjects. However, Astbury et al. [63] have shown that PDX is associated with higher GLP-1 and PYY responses and with lower ghrelin responses in lean men. The present study observed a trend in the decreasing levels of ghrelin and PYY with PDX consumption; however, the CCK concentration lacked any statistical significance due to vast variations recorded with the measurements. PYY is known to inhibit appetite and PDX has previously been demonstrated to increase acute satiety [39]. However, PYY also has other roles in appetite regulation [23, 69], and soluble fibers such as PDX do not necessarily have a similar effect on all satiety-promoting peptides. Endogenous ghrelin, on the other hand, stimulates gastric motility and appetite [23]. A recent animal study suggests that the SCFAs produced by colonic microbes as a result of the fermentation of dietary fiber can also cause L cells to trigger the release of GLP-1 and PYY [70]. This may also influence appetite by sustaining satiety after the meal and during the next meal.

It has been proposed that elevated triglyceride levels and an elevated expression of appetite hormones are related [71]. Obese individuals, in comparison to those of normal weight, have important differences in their expression of satiety hormones [14]. In fact, severely obese individuals have higher levels of leptin and lower levels of adiponectin [71] than those of normal weight. However, elevated levels to leptin have less impact on satiety in obese subjects [72]. Meier et al. [73] have proposed that GLP-1 abolishes the postprandial rise in triglyceride concentrations; therefore, our findings indicating that PDX increases the level of GLP-1 may be beneficial in reducing triglyceride lipids in obese individuals following a high energy meal.

Given the findings of decreased postprandial plasma lactate concentrations after the ingestion of the dietary supplemented PDX, this study brings new insights into obesity-linked postprandial metabolism. Obese subjects are known to have high plasma lactate levels caused by the increased lactate production in hypoxic adipose tissue [7, 8]. Lactate has several metabolic roles; it functions as an energy source and as a metabolic product in the muscle during intense exercise. It also induces insulin resistance in muscle cells [74] and stimulates inflammation in macrophages [75]. Lactate also modulates lipolysis in adipocytes [76], mediating the anti-lipolytic action of insulin [8, 76]. However, in the obese state an increase in lactate levels does not raise the anti-lipolytic effect of insulin because of the loss of insulin sensitivity [8]. This study shows the effect of dietary PDX on lower plasma lactate levels after the consumption of a high-fat meal. A lipid-containing meal is also reported to increase postprandial endotoxemia [77], and other recent clinical trials have also showed transient growth of circulating lipopolysaccharide (LPS) levels after the consumption of energy-rich meals (reviewed in [78]). In healthy humans the intravenous LPS treatment has also been shown to raise lactate levels within 90 minutes [79]. Both high-glucose and high-fat meals are reported to induce postprandial inflammation, while dietary fiber intake, in general, is associated with a reduced, low-grade inflammation [3].

Statistically significant changes were noted in the appetite ratings between the meals of this study, even though the power calculation was carried out according to the serum triglycerides (Tiihonen et al., unpublished observations). In this study, PDX reduced hunger by 40.4% as compared to the placebo during the satiety period. PDX also had a marginal effect on the feelings of satisfaction increasing them by 22.5% as compared to the placebo during the same period. Similar results have been observed previously regarding hunger [39]. However, this is the first time that this effect has been demonstrated with obese individuals. In addition, PDX has been shown to decrease energy intake in previous studies with normal weight and overweight participants [39, 40, 63]. These effects, however, were observed when PDX was administered as part of a mid-morning snack.

## Conclusion

The present study demonstrates that PDX induces an enhanced GLP-1 response after the ingestion of a high-fat meal by obese participants. GLP-1 secretion from the GI tract is known to decrease gastric emptying and to contribute to a decrease in food intake. PDX also induces a lower postprandial plasma lactate concentration, which can be associated with a lower post-meal inflammatory status. In addition, age seemed to have an effect on GLP-1 response showing lower GLP-1 levels with older participants. Finally, this study shows that PDX can decrease the subjective feelings of hunger. Thus, PDX may offer an additional way of regulating inter-meal satiety and improving postprandial metabolism in obese subjects.

## Abbreviations

BMI: Body mass index
CCK: Cholecystokinin
GI: Gastrointestinal
GLP-1: Glucagon-like peptide 1
HDL: High density lipoprotein
iAUC: Incremental area under the curve
LPS: Lipopolysaccharide
LDL: Low density lipoprotein
PYY: Peptide tyrosine tyrosine
PDX: Polydextrose
SFA: Saturated fatty acid
SCFA: Short chain fatty acid
SD: Standard deviation
SEM: Standard error of the mean
T2D: Type 2 diabetes
VAS: Visual analogue scales.
